# Supplementary material for: The cost-effectiveness of the Dutch In Balance fall prevention intervention compared to exercise recommendations among community-dwelling older adults with an increased risk of falls: A randomized controlled trial
Source: PLoS One. 2025 Dec 30;20(12):e0339497. doi: 10.1371/journal.pone.0339497 (PMC12752955; doi:10.1371/journal.pone.0339497)
Supplement: S1 Table — (DOCX) [file pone.0339497.s001.docx]

**S1: Calculation of the costs of the In Balance intervention**

| **Expenditures** | **Costs** |
| --- | --- |
| ***1 Information meeting (1.5 hours)*** | |
| Room rental | €50.00 |
| Therapist’s fee | €73.00 |
| Travel expenses for the therapist | €7.60 |
| Projector rental | €20.00 |
| Recruitment brochures | €30.00 |
| Press releases in local newspaper | €50.00 |
| Coffee | €20.00 |
| *Total information meeting* | *€250.60* |
|  | |
| ***3 Educational Meetings (2 hours)*** | |
| Room rental | €150.00 |
| Therapist’s fee | €219.00 |
| Travel expenses for the therapist | €22.80 |
| Course books | €144.00 |
| Coffee | €36.00 |
| *Total 3 educational meetings* | *€571.80* |
|  | |
| ***20 Training Sessions*** | |
| Room rental | €550.00 |
| Therapist’s fee | €949.00 |
| Travel expenses for the therapist | €167.20 |
| Coffee | €240.00 |
| *Total 20 training sessions* | *€1,906.20* |
|  | |
| Costs for organization, recruitment, and guidance | €292.00 |
|  | |
| ***Total costs*** | ***€3,020.60*** |
| ***Costs per participant*** | ***€252.00*** |
