# Supplementary material for: The cost-effectiveness of the Dutch In Balance fall prevention intervention compared to exercise recommendations among community-dwelling older adults with an increased risk of falls: A randomized controlled trial
Source: PLoS One. 2025 Dec 30;20(12):e0339497. doi: 10.1371/journal.pone.0339497 (PMC12752955; doi:10.1371/journal.pone.0339497)
Supplement: S2 Table — (DOCX) [file pone.0339497.s002.docx]

**S2: Baseline characteristics of the participants stratified for frailty status**

**Table 1. Baseline characteristics of the participants stratified for frailty status.** **Characteristics are presented as n (%) unless specified otherwise.**

| **Variable** | **Intervention group Non-frail (N = 40)** | **Intervention group Pre-frail (N = 91)** | **Control group Non-frail (N = 37)** | **Control group pre-frail (N = 96)** |
| --- | --- | --- | --- | --- |
| Age (years, SD) | 74.7 (5.2) | 75.4 (5.8) | 74.9 (5.0) | 75.9 (6.12) |
| Gender (female) | 27 (67.5%) | 72 (79.1%) | 26 (70.3%) | 76 (79.2%) |
| Body Mass Index (kg/m^2^, SD) | 25.2 (3.9) | 27.1 (4.2) | 26.2 (4.7) | 27.0 (5.1) |
| Mini-Mental State Examination (score, SD) | 28 (1.8) | 27.5 (2.3) | 27.4 (2.0) | 27.5 (2.4) |
| Marital status  Lawfully married/living together  Unmarried/divorced/widowed | 21 (53.8%)  18 (46.2%) | 37 (41.6%)  52 (58.4%) | 15 (41.7%)  21 (58.3%) | 46 (53.5%)  40 (46.5%) |
| Having children | 29 (74.4%) | 67 (75.3%) | 27 (75.0%) | 65 (73.9%) |
| Living alone | 20 (51.3%) | 52 (58.4%) | 21 (58.3%) | 45 (51.1%) |
| Education   Low  Moderate  High | 0 (0.0%) 9 (23.1%) 30 (76.9%) | 0 (0.0%) 29 (32.6%) 60 (67.4%) | 2 (5.6%) 6 (16.7%) 28 (77.8%) | 5 (5.7%) 25 (28.4%) 58 (65.9%) |
| Smoking | 3 (7.7%) | 3 (3.4%) | 2 (5.6%) | 5 (5.7%) |
| Use of alcohol | 28 (71.8%) | 63 (70.8%) | 28 (77.8%) | 64 (72.7%) |
| Use of different medications per week (SD) | 2.8 (4.8) | 3.3 (2.6) | 4.1 (9.1) | 4.3 (6.6) |
| Dizziness | 7 (17.9%) | 29 (32.6%) | 5 (15.2%) | 22 (26.5%) |
| Incontinence | 15 (38.5%) | 43 (48.3%) | 15 (41.7%) | 50 (56.8%) |
| How often fallen in previous year before start study  None/once  Twice or more | 26 (66.7%) 13 (33.3%) | 44 (50.6%) 43 (49.4%) | 23 (63.9%) 13 (36.1%) | 49 (55.7%) 39 (44.3%) |
| Use of aids   Walking  Vision  Hearing | 3 (7.7%) 39 (100%) 7 (17.9%) | 20 (22.5%) 87 (97.8%) 20 (22.5%) | 1 (2.8%) 34 (94.4%) 9 (25%) | 18 (20.5%) 84 (95.5%) 21 (23.9%) |
| Having physiotherapy | 8 (20.5%) | 36 (41.4%) | 10 (27.8%) | 30 (34.1%) |
| Physical activity per day (SD)  Number of hours being physically active  Number of steps | 1.67 (0.65)  7561.9 (3048.3) | 1.33 (0.65)  6424.7 (3509.6) | 1.47 (0.58)  7478.5 (3022.9) | 1.44 (0.69)  6668.9 (3499.3) |
| EQ-5D-5L Baseline utility (score, SD) | 0.87 (0.13) | 0.79 (0.13) | 0.87 (0.12) | 0.76 (0.16) |
| ASCOT Baseline utility (score, SD) | 0.91 (0.07) | 0.84 (0.15) | 0.90 (0.12) | 0.81 (0.15) |

Note. SD = Standard deviation, EQ-5D-5L = EuroQol questionnaire, ASCOT = Adult Social Care Outcome Toolkit questionnaire.
