# Supplementary material for: The cost-effectiveness of the Dutch In Balance fall prevention intervention compared to exercise recommendations among community-dwelling older adults with an increased risk of falls: A randomized controlled trial
Source: PLoS One. 2025 Dec 30;20(12):e0339497. doi: 10.1371/journal.pone.0339497 (PMC12752955; doi:10.1371/journal.pone.0339497)
Supplement: S3 Table — (DOCX) [file pone.0339497.s003.docx]

**S3: Baseline characteristics of the participants per-protocol**

**Table 1. Baseline characteristics of the participants per-protocol. Characteristics are presented as n (%) unless specified otherwise.**

| **Variable** | **Intervention group (N = 54)** | **Control group (N = 130)** |
| --- | --- | --- |
| Age (years, SD) | 75.9 (6.4) | 75.5 (5.8) |
| Gender (female) | 40 (74.1%) | 100 (76.9%) |
| Body Mass Index (kg/m^2^, SD) | 25.9 (4.1) | 26.8 (5.0) |
| Mini-Mental State Examination (score, SD) | 27.6 (2.1) | 27.4 (2.3) |
| Frailty status (pre-frail) | 41 (75.9%) | 93 (71.5%) |
| Marital status  Lawfully married/living together  Unmarried/divorced/widowed | 26 (48.1%)  28 (51.9%) | 58 (48.7%)  61 (51.3%) |
| Having children | 37 (68.5%) | 90 (74.4%) |
| Living alone | 28 (51.9%) | 66 (54.5%) |
| Education   Low  Moderate  High | 0 (0.0%) 17 (31.5%) 37 (68.5%) | 7 (5.8%) 31 (25.6%) 83 (68.6%) |
| Smoking | 1 (1.9%) | 7 (5.8%) |
| Use of alcohol | 40 (74.1%) | 91 (75.2%) |
| Use of different medications per week (SD) | 3.7 (4.4) | 4.2 (7.4) |
| Dizziness | 16 (29.6%) | 27 (23.9%) |
| Incontinence | 29 (53.7%) | 62 (51.2%) |
| How often fallen in previous year before start study  None/once  Twice or more | 24 (45.3%) 29 (54.7%) | 70 (57.9%) 51 (42.1%) |
| Use of aids  Walking  Vision  Hearing | 12 (22.2%) 53 (98.1%) 14 (25.9%) | 17 (14.0%) 116 (95.9%) 29 (24.0%) |
| Having physiotherapy | 20 (37.0%) | 38 (31.4%) |
| Physical activity per day (SD)  Number of hours being physically active  Number of steps | 1.32 (0.57) 6947.4 (3380.8) | 1.46 (0.66) 6230.3 (2794.3) |
| EQ-5D-5L Baseline utility (score, SD) | 0.81 (0.14) | 0.79 (0.16) |
| ASCOT Baseline utility (score, SD) | 0.88 (0.11) | 0.84 (0.15) |

Note. SD = Standard deviation, EQ-5D-5L = EuroQol questionnaire, ASCOT = Adult Social Care Outcome Toolkit questionnaire.
