# Supplementary figures and images for: The cost-effectiveness of the Dutch In Balance fall prevention intervention compared to exercise recommendations among community-dwelling older adults with an increased risk of falls: A randomized controlled trial
Source: PLoS One. 2025 Dec 30;20(12):e0339497. doi: 10.1371/journal.pone.0339497 (PMC12752955; doi:10.1371/journal.pone.0339497)

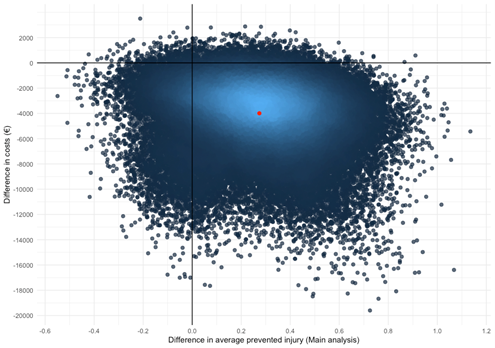

Supplement: S4 Fig — (TIF) [file pone.0339497.s004.tif]

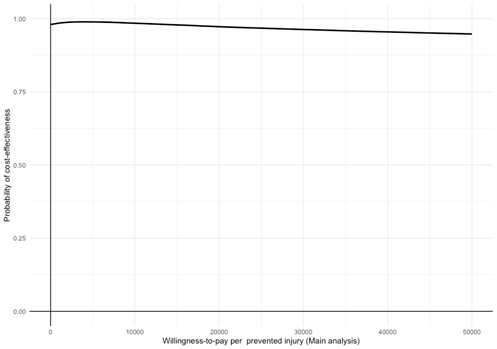

Supplement: S4 Fig — (TIF) [file pone.0339497.s005.tif]

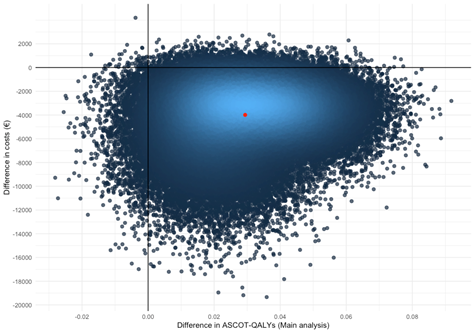

Supplement: S5 Fig — (TIF) [file pone.0339497.s006.tif]

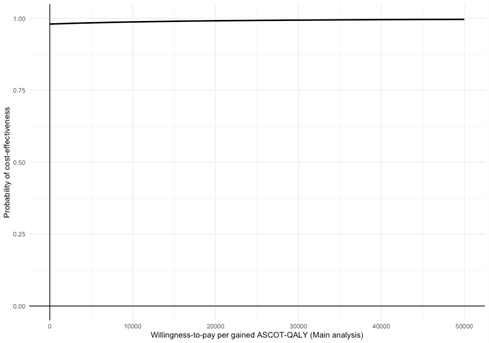

Supplement: S5 Fig — (TIF) [file pone.0339497.s007.tif]
