# Supplementary material for: The cost-effectiveness of the Dutch In Balance fall prevention intervention compared to exercise recommendations among community-dwelling older adults with an increased risk of falls: A randomized controlled trial
Source: PLoS One. 2025 Dec 30;20(12):e0339497. doi: 10.1371/journal.pone.0339497 (PMC12752955; doi:10.1371/journal.pone.0339497)
Supplement: S1 Protocol — (PDF) [file pone.0339497.s008.pdf]

In Balans

In Balans: de (kosten-)effectiviteit van de In Balans valpreventietraining op vallen en valletsels bij thuiswonende ouderen met een verhoogd valrisico

PROTOCOL TITLE 'In Balans: the (cost-)effectiveness of the In Balans fall prevention training on falls and fall injuries in community-dwelling older adults with an increased risk of falls.'

**Protocol ID**	**50-55510-98-086**
**Short title**	**In Balans**
**EudraCT number**	**Not applicable**
**Version**	**1**
**Date**	**28-10-2020**
**Coordinating investigator/project leader**	**Prof. dr. M. Pijnappels, Department of Human Movement Sciences, Vrije Universiteit Amsterdam**
**Principal investigator**	**Prof. dr. M. Pijnappels, Department of Human Movement Sciences, Vrije Universiteit Amsterdam**
**Uitvoerend onderzoeker**<br><br>**Multicenter research: per site**	**Maaike van Gameren, PhD student, Department of Human Movement Sciences, Vrije Universiteit Amsterdam**<br><br>**Prof. dr. M. Pijnappels, Department of Human Movement Sciences, Vrije Universiteit Amsterdam**
**Sponsor (in Dutch: verrichter/opdrachtgever)**	**Vrije Universiteit Amsterdam, Department of Human Movement Sciences**
**Subsidising party**	**ZonMw-Effectonderzoek Lacunes**
**Independent expert (s)**	**Prof. dr. Nathalie van der Velde, Department of Geriatric Medicine, Amsterdam UMC, location AMC**
**Laboratory sites**	**Not applicable**
**Pharmacy**	**Not applicable**

PROTOCOL SIGNATURE SHEET

Name	Signature	Date
**Head of Department :**<br>***Prof. dr. Jaap van Dieën***<br>*Department of Human Movement Sciences, Vrije Universiteit Amsterdam*		
**Coordinating Investigator/Project leader/Principal Investigator:**<br>***Prof. dr. Mirjam Pijnappels***<br>*Department of Human Movement Sciences, Vrije Universiteit Amsterdam*		

TABLE OF CONTENTS

INTRODUCTION AND RATIONALE 8
1.1 FALLS IN OLDER ADULTS 8
1.2 FALL PREVENTION PROGRAMMES 8
1.3 'IN BALANS' PROGRAMME 8
1.4 RATIONALE OF THIS STUDY 9
1.4.1 Relevance from the societal perspective 9
1.4.2 Relevance from the intervention perspective 9
2. OBJECTIVES 10
3. STUDY DESIGN 10
4. STUDY POPULATION 10
4.1 Population 10
4.2 Inclusion criteria 10
4.3 Exclusion criteria 11
4.4 Sample size calculation 11
5. TREATMENT OF SUBJECTS 11
5.1 Investigational product/treatment 11
5.2 Use of co-intervention 12
5.3 Escape medication 12
6. METHODS 12
6.1 Study parameters/endpoints 12
6.1.1 Main study parameter/endpoint 12
6.1.2 Secondary study parameters/endpoints 13
6.1.3 Other study parameters 14
6.2 Randomisation, blinding and treatment allocation 14
6.3 Study procedures 14
6.4 Withdrawal of individual subjects 15
6.5 Replacement of individual subjects after withdrawal 15
6.6 Follow-up of subjects withdrawn from treatment 15
6.7 Premature termination of the study 15
7. SAFETY REPORTING 15
7.1 Temporary halt for reasons of subject safety 15
7.2 AEs, SAEs and SUSARs 16
7.2.1 Adverse events (AEs) 16
7.2.2 Serious adverse events (SAEs) 16
7.2.3 Suspected unexpected serious adverse reactions (SUSARs) 16
7.3 Annual safety report 16
7.4 Follow-up of adverse events 16
7.5 Data Safety Monitoring Board (DSMB) / Safety Committee 16
8. STATISTICAL ANALYSIS 17
8.1 Primary study parameter(s) 17
8.2 Secondary study parameter(s) 18

8.3 Other study parameters 18
8.4 Interim analysis (if applicable) 18
9. ETHICAL CONSIDERATIONS 18
9.1 Regulation statement 18
9.2 Recruitment and consent 18
9.3 Objection by minors or incapacitated subjects (if applicable) 19
9.4 Benefits and risks assessment, group relatedness 19
9.5 Compensation for injury 19
9.6 Incentives (if applicable) 19
10. ADMINISTRATIVE ASPECTS, MONITORING AND PUBLICATION 19
10.1 Handling and storage of data and documents 19
10.2 Monitoring and Quality Assurance 20
10.3 Amendments 20
10.4 Annual progress report 20
10.5 Temporary halt and (prematurely) end of study report 20
10.6 Public disclosure and publication policy 20
11. REFERENCES 21

LIST OF ABBREVIATIONS AND RELEVANT DEFINITIONS

AE	Adverse Event
BIA	Budget impact analysis
BMI	Body mass index
CCMO	Central Committee on Research Involving Human Subjects; in Dutch: Centrale Commissie Mensgebonden Onderzoek
DSMB	Data Safety Monitoring Board
IC	Informed Consent
ICER	Incremental cost effectiveness ratio
METC	Medical research ethics committee (MREC); in Dutch: medisch-ethische toetsingscommissie (METC)
RCT	Randomized controlled trial
(S)AE	(Serious) Adverse Event
Sponsor	The sponsor is the party that commissions the organisation or performance of the research, for example a pharmaceutical<br>company, academic hospital, scientific organisation or investigator. A party that provides funding for a study but does not commission it is not regarded as the sponsor, but referred to as a subsidising party.
WMO	Medical Research Involving Human Subjects Act; in Dutch: Wet Medisch-wetenschappelijk Onderzoek met Mensen

SUMMARY

Rationale: Falls and fall related injuries are a serious threat to life expectancy and quality of life in our ageing population. Moreover, they may be related to high healthcare and societal costs. Therefore, it is important to implement fall prevention interventions that older adults can continue themselves. 'In Balans' is a 14-week, low-cost group intervention, that is widely used in pre-frail older adults with an increased risk of falling. One previous study of Faber et al. in a nursing home population found that 'In Balans' resulted in a fall risk reduction of 61% in pre-frail but not in frail older adults (1). Now it is time to fill the knowledge gap on the (cost-)effectiveness of 'In Balans' for pre-frail and non-frail older adults with an increased fall risk living in the community.
Objective: The aim of this study is to assess the (cost-)effectiveness of the fall prevention intervention 'In Balans' in community-dwelling older adults with an increased risk of falls compared to general exercise recommendations.
Study design: A single-blinded, multicenter randomized controlled trial.
Study population: 256 pre-frail and non-frail older adults (≥65 years old) with an increased risk of falls.
Intervention: 'In Balans' is a 14-week group exercise programme. The first four weeks include counselling and education meetings with topics regarding fall prevention. The last ten weeks contain two exercise meetings per week. Exercises are derived from principles of Tai Chi and are mainly focused on balance and strength.
Comparator: Written general exercise recommendations on physical activity levels, strength and balance according to the Dutch Guidelines for Physical Activity.
Main study parameters/endpoints: The number of falls (with and without injuries) over 12 months.
Nature and extent of the burden and risks associated with participation, benefit and group relatedness: The assessments (baseline, 4 and 12 months) consist of questionnaires, physical performance measures and 1-week activity monitoring. Participants will also be asked to fill in a monthly fall calendar. This does not interfere with daily living and has no risks involved. The physical assessments will be executed in a private controlled setting. It is assumed that participants benefit from the intervention since the exercises will improve balance, mobility and self-confidence. These improvements may lead to less risk of falling, less fear of falling and therefore to a higher quality of life. The group sessions take place under supervision of trained and certified physical therapists (Physiotherapists or Exercise therapists) who will ensure the safety of the participants.

INTRODUCTION AND RATIONALE

1.1 FALLS IN OLDER ADULTS

The Dutch population is ageing. About 19% of the population is currently 65 years or older; this percentage will increase to 24% (i.e. 4 million older adults) in 2030 (2). This trend puts pressure on our healthcare system because of an increased need for healthcare and long term care services. Therefore, the government stimulates prevention activities for self-management among older adults. One of the main opportunities for prevention is fall prevention. Approximately 30% of older adults over 65 years of age fall once per year and this percentage increases with age. A fall is the number one cause of injury in older adults. In 2018, 108,000 older adults (one every 5 minutes) visited an Emergency Department due to a fall-related injury and 4,396 older adults died as a result of a fall, with total healthcare costs estimated at €960 million (3). The first fall of an older person is a critical starting point of a cascade of events that can lead to injury, inactivity, other health problems, social isolation, more falls and institutionalisation. The alarming impact of falls on older individuals, healthcare and society in our greying society renders the prevention of falls and fall related injuries most timely and highly urgent.

1.2 FALL PREVENTION PROGRAMMES

A recent systematic review showed that exercise programmes are effective interventions to prevent falls (4). Group interventions seem more effective than individual programmes, which may be related to the fact that compliance is much lower for individual programmes (5). Several fall prevention exercise programmes have been described, evaluated and implemented in the Netherlands (6); they all aim to prevent falls, but use different approaches and aim at different target groups (7). ‘Zicht op evenwicht’ is a cognitive intervention to reduce fear of falling; it does not contain exercise components and has been shown effective to reduce fear and activity avoidance in people with fear of falling and anxiety (8). The ‘Otago’ exercise programme (either individual or in a group) has strong evidence for effectiveness and has been shown most suitable for frail older adults (9). ‘Vallen Verleden Tijd’ is a group exercise intervention, with strong evidence for effectiveness (10, 11), that consists of complex and challenging exercises (obstacle pathways) and a focus on learning fall techniques. This intervention is most suitable for relatively fit older adults (with and without fall risk) and requires an extensive course for trainers and a substantial amount of materials. Consequently, many older adults are not eligible for Vallen Verleden Tijd and widespread implementation may not be feasible.

1.3 ‘IN BALANS’ PROGRAMME

‘In Balans' is a 14-week group exercise programme, with the aim to reduce falls by increasing awareness, balance and strength in older (pre-frail) individuals at risk of falling. The first four weeks include counselling and education meetings on topics regarding fall prevention. The last ten weeks contain two exercise meetings per week. Exercises are derived from principles of Tai Chi and are mainly focused on balance and strength. One previous RCT in pre-frail and frail older adults living in a nursing home, showed a strong reduction (61%) in the risk of becoming a faller in the pre-frail group only (1). Since this study is conducted, several elements have been changed (e.g. the composition of the target group and the duration of the programme). ‘In Balans’ is nationally well-known by healthcare professionals, insurance companies, municipalities and older adults. It is a commonly used fall prevention programme as it requires only minimal equipment (in contrast to other programmes such as 'Vallen Verleden Tijd') and is, therefore, relatively cheap to implement. Over the past years, a large

number of therapists have been trained and are certified to provide the 'In Balans' intervention. In Balans is widely practiced, as is shown by the number of ‘In Balans’ trainers and sold instruction books. Between 2016 and 2018, 464 professionals were trained to become an ‘In Balans’ trainer. In 2017, 739 and in 2018, 1059 instruction books were sold, which is an indication of how many older adults participated in an ‘In Balans’ training.

1.4 RATIONALE OF THIS STUDY

Several evaluations (12, 13) showed that older adults appreciate the present content of the ‘In Balans’ program and professionals indicate that they notice improvements in physical functioning among older adults who completed the ‘In Balans’ program. However, these evaluations were not conducted using a rigorous design, such as a RCT. Hence, it is essential to assess the (cost-)effectiveness of ‘In Balans’ in its current form to reduce the number of falls (with and without injuries) for an extended target group (pre-frail as well as non-frail individuals with an increased fall risk living in the community), and to evaluate the effects that are sustained over a longer follow-up period. The rationale of this study can be motivated from two main perspectives.

1.4.1 Relevance from the societal perspective

According to Statistics Netherlands (CBS), the number of people aged over 65 years has risen to almost 3.2 million in 2019 (2). A previous study has shown that approximately 20-25% of these older adults can be defined as pre-frail, with approximately 70% having problems in physical functioning (14). It is estimated that every year about one third of the older adult population will fall. The introduction of geriatric physiotherapy and an increased focus on evidence based practice, cause an increasing demand for evidence on the effectiveness of interventions. From conversations with trainers it appears that trainers are willing to use the program ‘In Balans’, but are critical regarding the effectiveness of the intervention, as it was last scientifically studied in 2006. Moreover, health insurance companies require evidence on the cost-effectiveness of In Balans in order to decide whether the intervention becomes or stays part of insured healthcare.

1.4.2 Relevance from the intervention perspective

The effectiveness of ‘In Balans’ among pre-frail older adults has been demonstrated by Faber et al (1). The target group of ‘In Balans’ (older adults of 65 year or older with a risk of falling) has changed since then. In 2006, this group mainly lived in nursing homes. Nowadays, most people in this group live independently. Furthermore, the intervention in its current form is provided to a broad range of older adults with respect to age, gender, education level or mastery of language. Also, based on previous experiences, the intervention is shortened from 20 weeks to 14 weeks which may have influenced the effectiveness of ‘In Balans’. For healthcare professionals, researchers, and policy makers, who work with and facilitate ‘In Balans’, it is of great importance to investigate the (cost-)effectiveness of the current intervention in this new target group. This will help to efficiently allocate the resources available, as this is currently one of the major bottlenecks for implementation.

2. OBJECTIVES

Primary aim:

1. To evaluate post-intervention (4 months) and long term (12 months) effects of the 'In Balans' intervention in community-dwelling older persons (≥65 years) with an increased risk of falls on the number of falls (with and without injuries), compared to general exercise recommendations.

Secondary aims

2. To evaluate the cost-effectiveness of the 'In Balans' intervention compared to general exercise recommendations from a societal perspective.
3. To assess the effects of the 'In Balans' intervention on the secondary outcome measures physical performance, physical activity level, perceived confidence, quality of life and empowerment compared to general exercise recommendations.
4. To assess the difference in 'In Balans' intervention effectiveness between pre-frail and non-frail community-dwelling older persons in all outcome measures described in aim 1, 2 and 3.
5. To evaluate the facilitators and barriers that affect the implementation of the 'In Balans' intervention in practice.

3. STUDY DESIGN

This study is a single-blinded, multicenter randomized controlled trial with stratified randomization for frailty at the subject level (for an overview of the study procedure see figure 1). Participants and 'In Balans' trainers will not be blinded because of the nature of the treatment strategies. Participants from the community setting will be randomized into the intervention ('In Balans') or control condition (general exercise recommendations). All participants will be followed for one year. Their physical performance will be measured and participants will be asked to complete questionnaires at baseline, and 4, 8 and 12 months follow-up. In addition, for implementing recommendations 3 focus groups will be conducted with participants, trainers and other stakeholders.

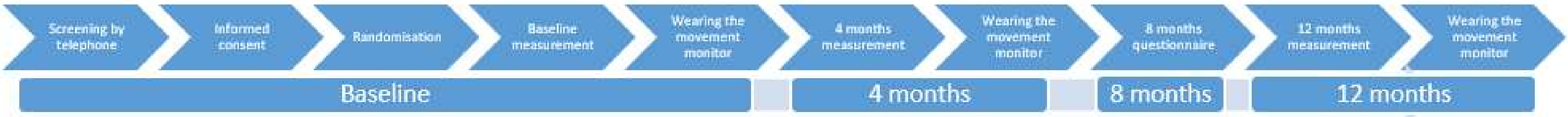

Figure 1. Overview of the study procedure

4. STUDY POPULATION

4.1 Population

Community-dwelling older adults with an increased risk of falls.

4.2 Inclusion criteria

To be eligible for participating in this study, a person should meet all of the following criteria:

- Aged 65 years or older
- Have a potential fall risk, as assessed by the 'fall risk screening questionnaire' (participants meet one of the following criteria: having a history of falls in the past 12 months and/or having difficulties with walking, balance and/or mobility) (15, 16)

4.3 Exclusion criteria

People that meet any of the following criteria will be excluded from participation in this study:

- Younger than 65 years of age
- Mini-mental state examination of <19/30
- Classified as frail based on the 'Groningen Frailty Indicator' (17) or the phonotype concept introduced by Fried et al. in which 3 or more points will be considered as frail (18)
- Not able to read or understand Dutch
- People with contra-indications that make it impossible to participate in the In Balans program are excluded (e.g. cardiovascular, neurological, orthopedic problems)

4.4 Sample size calculation

The target population in this study is pre-frail older adults (≥65 years). In the Netherlands approximately 20-25% of the older adults is pre-frail (14). In the general population of older adults, about 1 out of 3 persons (33%) falls at least once per year (19). To obtain a reduction of 50% in the number of falls between the intervention and control group, a minimum of 106 persons are required per group, at a power of 0.80, beta of 0.02 and alpha of 0.05. Taking into account a dropout rate of 20%, the required sample size is 128 participants per intervention and control group. Hence, 11 to 14 'In Balans' intervention groups, of 9-12 participants each, are needed to include the required sample size of 256 participants.

5. TREATMENT OF SUBJECTS

5.1 Investigational product/treatment

Intervention

'In Balans' is a 14-week group intervention for older adults at increased risk of falls. It combines educational and exercise components to raise awareness of fall risk factors, to improve balance and mobility, and overall to increase self-confidence. The intervention consists of three phases. The first phase (week 1) comprises one information meeting about the impact a fall can have on someone's life and the purpose of 'In Balans'. In the second phase (week 2-4), there are three weekly educational meetings, where the following topics are addressed and discussed: increasing awareness of one's fall risk and balance disturbance, increasing knowledge about how to cope with effective fall prevention methods and getting acquainted with the upcoming training weeks. The third phase (week 5-14) is an exercise program of 10 weeks, with two training sessions per week for the duration of one hour. Exercises are derived from principles of Tai Chi, with balance and strength elements and with emphasis on standing strong and shifting weight. In this study the intervention will be given by trained and certified physical therapists and exercise therapists (20).

Control group

The control group will receive written general exercise recommendations on physical activity levels, strength and balance according to the Dutch Guidelines for Physical Activity (21). The content of these written materials will include the following recommendations:

"Engage in moderately intensive physical activity such as walking or cycling for at least 150 minutes a week, spread over several days. The longer you are physically active, and the more frequently and/or more vigorous the activity, the greater the health benefit derived. Some physical activity is better than none." (21)

"Engage in physical activities that strengthen muscles and bones at least twice a week, such as climbing stairs, repeatedly rising from your chair, and strength training, and combine these with balance exercises." (21)

5.2 Use of co-intervention

Participants in both trial arms are allowed to use any co-intervention, including analgesics, and referral to other health care professionals.

5.3 Escape medication

Not applicable

6. METHODS

6.1 Study parameters/endpoints

Participants will be assessed at three moments in time; at baseline (M0, before randomization) and at 4 months (M4, after completion of the intervention, short term effects) and 12 months (M12, long term effects) follow up. The assessments at these three measurement points will consist of questionnaires, physical performance measures and 1-week activity monitoring. For the economic evaluation, information will be collected on the subject's quality of life, healthcare utilization, own expenses and productivity losses with three retrospective 4-month cost questionnaires at M4, M8 and M12. For the process evaluation and recommendations for implementation, attendance to the training will be monitored, and a small number of process evaluation questions will be added to the questionnaires for the 'In Balans' participants at 4 months. Furthermore, focus groups will be conducted with 1) older adults (study participants of the In Balans group), 2) 'In Balans' trainers and 3) other stakeholders to obtain facilitating and hindering factors for the implementation of the 'In Balans' intervention after 12 months.

6.1.1 Main study parameter/endpoint

The primary outcome measure in this study is the <u>number of falls</u> (with or without injury) measured with fall diaries and monthly follow-up telephone calls. This combination of pro- and retrospective data collection follows recommended guidelines for conducting falls prevention trials (22). Participants will be asked to record fall and fracture events every week on the 'fall and fracture calendar'. Each month the calendars will be send and collected by the research team. In the telephone survey the participant will be asked about falls that occurred in the previous month. If the participant responds with "no" or "I don't know/remember", the survey is complete. Only respondents who answer with "yes" will be asked: "How many times did you fall over the past month?" and "did you suffer from any injuries resulted by the fall or falls" "What type of injury do/did you have?". For an overview of the measurements see attachment 1.

6.1.2 Secondary study parameters/endpoints

Economic outcome measures (assessed at M4, M8, M12)

- Quality of Life assessed with the five-level version of the EuroQol 5D (EQ-5D-5L) and the Adult social care outcomes toolkit (ASCOT). The EQ-5D-5L contains 5 dimensions for measuring generic health status in order to calculate quality-adjusted life years (QALY) (23). The ASCOT contains 9 questions for measuring social care-related quality of life (24).
- Healthcare, subject and family costs: the iMTA Medical Cost Questionnaire (iMCQ) with a recall period of 4 months. This instruments contains 7 questions and measures healthcare utilization (25).
- Productivity costs: the iMTA Productivity Cost Questionnaire (iPCQ) with a recall period of 4 months. It includes 10 items. Three modules measuring productivity losses of paid work due to 1) absenteeism and 2) presenteeism and productivity losses related to 3) unpaid work. The questionnaire adopts a recall period of 4 months (26).

Performance based physical functioning (assessed at M0, M4, M12)

- Balance measured with the 'Performance-Oriented Mobility Assessment – Balance' (27), the 'Four Stage Balance Test' (28) and the 'Timed Up and Go Test' (29)
- Mobility measured with the 'Performance-Oriented Mobility Assessment – Mobility' (27)
- Muscle Strength measured with the 'Hand Grip Strength Test' (30), the 'Timed Chair Stands Test' (31) and the 'Short Physical Performance Battery' (32).
- Walking Speed measured with the '10 Meter Walk Test' (33)
- Aerobic Endurance measured with the '2-minute Step Test' (34)
- Fall Risk measured with the 'LASA Fall Risk Questionnaire' (35)
- Daily Physical Activity and Gait Pattern, as assessed with an inertial sensor, worn on the lower back for the duration of one week (36, 37)

Questionnaires (assessed at M0, M4, M12)

- General Health Status with the 'Short Form Health Survey - SF-36' (38) and 'Positive Health Inventory Tool' (39)
- Empowerment with the 'Pearlin Mastery Scale' (40) and 'General Self-Efficacy Scale' (41)
- Fear of Falling with the 'Falls Efficacy Scale International -FES-I' (42)
- Walking Confidence with the 'Modified Gait Efficacy Scale – mGES' (43)
- Causes (behavioral, biological, environmental), circumstances and consequences (none, minor, moderate, major of falls occurring during follow up, with the fall calendar and monthly telephone calls (44).

Process evaluation

In addition to the effectiveness study, a process evaluation will be performed. The process evaluation will provide us information about facilitating and hindering factors for implementation and sustainable use of 'In Balans' in practice, which can provide suggestions for implementation strategies and intervention improvements.

- Process evaluation questions will be included in the questionnaire for the intervention group (M4). These questions examine participants' experiences with the 'In Balans' program.
- Adherence will be measured by the number of actual sessions per group and number of training sessions per participant (attendance list) provided by 'In Balans' trainers in the intervention group.
- 3 focus groups with 10-15 participants, 5 'In Balans' trainers and 6 other stakeholders (municipality, health care insurance company, community health service, KNGF, VvOCM, KBO-PCOB) at the end of the RCT (M12).

6.1.3 Other study parameters

Demographic measures (MO and M12)

- Age, gender, marital status, living situation, smoking, fall history and presence of chronic diseases.
- Frailty Status will be assessed using the 'Groningen Frailty Indicator- GFI' (17) and '5 Frailty Indicators': weight (loss), weakness, exhaustion, slowness, low physical activity (18).

6.2 Randomisation, blinding and treatment allocation

Eligible participants will be randomized by an independent researcher directly after the baseline assessments into the intervention group ('In Balans') or control group (general exercise recommendations). Participants will be randomized according to a stratified randomization protocol by using a computer-generated sequence table. In order to balance the size of the intervention and control groups, randomization will be stratified according to frailty. Based on the indicators of Fried et al. (18), participants with 0 indicators will be categorized as non-frail; participants with 1 or 2 indicators will be categorized as pre-frail. The aim is to include 30-50% participants in the pre-frail subgroup and 50-70% participants in the non-frail subgroup in both control and intervention group. All participants will be given a unique identification number. Participants will be informed by e-mail or a letter about their allocation. In Balans trainers and community-dwelling older adults will not be blinded to group allocation. The principal investigator will be blinded to group assignment until completion of statistical analyses. The assessors (physiotherapy or exercise therapy students), will also be blinded to group allocation.

6.3 Study procedures

Participants will be recruited through flyers, advertisements and personal letters via general practitioners in neighbourhoods of 7-14 certified and registered 'In Balans' trainers in 2-4 municipalities across the Netherlands. In addition, participants will be recruited at annual flu shots and through flyers, folders at supermarkets, pharmacists, GP practices and community centres, and advertisements in local papers. Previous experiences have shown that such an approach with personal letters and flyers is effective in recruiting the specific target group. Persons who are interested in participating will be contacted by telephone for eligibility screening. Persons will be excluded during the telephone screening if they a) are younger than 65 years of age, b) have no increased fall risk, as assessed by the 'fall risk screening instrument' (15), c) are classified as frail, based on the 'Groningen Frailty Indicator' (45) and d) are not able to read or understand Dutch. Those who are eligible will receive an extended information letter, in which the study procedure, including randomization, will be explained. They will be asked for their informed consent for participation in the study. After informed consent, as part of the baseline measurement, participants will be assessed on their cognition and frailty status and will be excluded if they a) have indications for severe cognitive impairment

(MiniMental State Examination < 19/30), b) have 3 or more out of 5 physical frailty criteria, according to Fried ea. (18). These physical frailty criteria include *Weight* (Body Mass Index <18.5 kg/m^2), *Weakness* (sex and body mass specific grip strength), *Exhaustion* (SF-36 vitality score <75), *Slowness*: (gender and height specific gait speed), and *low physical activity levels* (SQUASH questionnaire). After the baseline assessments, participants will be randomized stratified for frailty status (1:2 pre-frail versus non-frail). In total, participants will be measured at three moments: before randomization (baseline), after 4 and 12 months. The assessments will consist of online questionnaires, accelerometers and performance tests. A reminder will be send to participants who do not fill in the questionnaires within 1 week. If this action does not lead to completing the missing questionnaire, the primary researcher (MvG) and/or research assistant contacts the participant by phone for a final request to complete the questionnaire. Participants will receive an accelerometer for the measurement of objective physical activity. Performance based measurements will be conducted by trained research assistants who will not be involved in the intervention and will be blinded to group allocation. To evaluate the cost-effectiveness of the intervention, information on the subjects' healthcare utilization and productivity losses will be gathered with three retrospective 4-month cost questionnaires. In addition to the quantitative effectiveness study, three focus groups with participants, In Balans trainers and other stake holders will be held after 12 months.

6.4 Withdrawal of individual subjects

Subjects can leave the study at any time for any reason if they wish to do so without any consequences. The investigator can decide to withdraw a subject from the study for urgent medical reasons.

6.5 Replacement of individual subjects after withdrawal

Withdrawn subjects will not be replaced.

6.6 Follow-up of subjects withdrawn from treatment

Participants who decide to stop with the 'In Balans' program, will still be asked to complete the follow-up questionnaires.

6.7 Premature termination of the study

Any of the following conditions leads to premature termination:

- Request by subject to discontinue for any reason during the study (withdrawal of consent);
- Adverse event or illness that, in the opinion of the investigator, warrants the participant's withdrawal from intervention;
- If, according the independent study physician or study team, continuation of the study will lead to increased health risks;
- Death of the participant;
- Participants who do not follow the requirements of the investigator, especially those concerning safety and/or if the participant after his enrolment is uncooperative or not willing to comply with the protocol (non-compliant);

7. SAFETY REPORTING

7.1 Temporary halt for reasons of subject safety

In accordance to section 10, subsection 4, of the WMO, the sponsor will suspend the study if there is sufficient ground that continuation of the study will jeopardise subject health or safety. The sponsor will notify the accredited METC without undue delay of a temporary halt including the reason for such

an action. The study will be suspended pending a further positive decision by the accredited METC. The investigator will take care that all subjects are kept informed.

7.2 AEs, SAEs and SUSARs

7.2.1 Adverse events (AEs)

Adverse events are defined as any undesirable experience occurring to a subject during the study, whether or not considered related to the 'In Balans' intervention or the trial procedure. All adverse events reported spontaneously by the subject or observed by the investigator or his staff will be recorded. Occurrence of AEs will be assessed during the monthly telephone calls.

7.2.2 Serious adverse events (SAEs)

SAEs will be reported by the In Balans trainers, participant, family or involved researchers according to Art. 1 lid 1q WMO. Occurrence of SAEs will be assessed during the monthly telephone calls by the first investigator (MvG) or reported to the coordinating investigator (MP). The sponsor will report the SAEs through the web portal *ToetsingOnline* to the accredited METC that approved the protocol, within 7 days of first knowledge for SAEs that result in death or are life threatening followed by a period of maximum of 8 days to complete the initial preliminary report. All other SAEs will be reported within a period of maximum 15 days after the sponsor has first knowledge of the serious adverse events.

A serious adverse event is any untoward medical occurrence or effect that

- Results in death;
- Is life threatening (at the time of the event);
- Requires hospitalisation or prolongation of existing inpatients' hospitalisation;
- Results in persistent or significant disability or incapacity;
- Is a congenital anomaly or birth defect;
- Any other important medical event that did not result in any of the outcomes listed above due medical or surgical intervention but could have been based upon appropriate judgement by the investigator.

An elective hospital admission will not be considered as a serious adverse event.

7.2.3 Suspected unexpected serious adverse reactions (SUSARs)

Not applicable

7.3 Annual safety report

Not applicable

7.4 Follow-up of adverse events

All AEs will be followed until they have abated, or until a stable situation has been reached. Depending on the event, follow up may require additional tests or medical procedures as indicated, and/or referral to the general physician or a medical specialist. SAEs need to be reported till the end of the study within the Netherlands, as defined in the protocol.

7.5 Data Safety Monitoring Board (DSMB) / Safety Committee

A DSMB is not installed given the low safety risks for the participants. The study does not investigate and medicinal product, procedures are not invasive and we will not focus on severe outcome

measures (e.g. dead or severe injuries).

8. STATISTICAL ANALYSIS

Effect evaluation

Descriptive analyses will be used to establish mean scores, standard deviations and to identify outliers. Statistical analyses will be performed according to the intention-to-treat principle. The main analysis will be conducted for the whole study population. However, to identify possible differences in intervention effects between non-frail and pre-frail respondents, subgroup analyses will be performed, stratified for frailty level. Differences between the intervention group and control group on the primary and secondary outcomes will be analyzed with multilevel (mixed model) regression analyses. Three hierarchical levels will be included in the mixed models; 'In Balans' trainers, subject and time. If necessary, analyses will be adjusted for confounders and/or stratified for effect modifiers. Secondary outcomes will be analyzed in a similar fashion.

Economic evaluation

All statistical analyses will be done according to the intention-to treat principle. For the economic evaluation, missing cost and effect data will be imputed using multiple imputation according to the MICE algorithm developed by van Buuren et al. (46). Rubin's rules will be used to pool the results from the different multiply imputed datasets. Linear regression analyses will be used to estimate cost and effect differences between intervention and control while adjusting for confounders if necessary. Incremental cost-effectiveness ratios (ICERs) will be calculated by dividing the difference in the mean total costs between the treatment groups by the difference in mean effects between the treatment groups. Bias-corrected and accelerated bootstrapping with 5000 replications will be used to estimate 95% confidence intervals around the cost differences and statistical uncertainty surrounding the ICERs. Uncertainty surrounding the ICERs will be graphically presented on cost-effectiveness planes. Cost-effectiveness acceptability curves will also be estimated showing the probability that the intervention is cost-effective in comparison with control for a range of different ceiling ratios thereby showing decision uncertainty (47). A budget impact analysis (BIA) will be conducted from the perspective of health-care decision makers according to the Dutch guidelines (48) and the recommendations from Sullivan ea. (49). In the BIA, data from the effectiveness and cost-effectiveness analyses regarding the differences in costs and health outcomes will be combined with national prevalence and incidence data to extrapolate the findings to a time horizon of 5 years.

Process evaluation

For the process evaluation, a small number of process evaluation questions will be added to the questionnaires for participants of the 'In Balans' intervention group, at 4 months follow-up and monitor the monitor the content of the intervention provided to participants. In addition, three focus groups will be performed in which participants' and stakeholders' experiences with 'In Balans' will be discussed in order to develop recommendations for implementation.

8.1 Primary study parameter(s)

The primary outcome of the study is the number of falls. The primary effect in these analyses is described by the coefficient of the time treatment interaction term (continuous outcome variable). Differences in the change scores between the intervention group and control group will be analyzed with multilevel (mixed model) linear regression analyses. Three hierarchical levels will be included in

this analysis; 1) 'In Balans' trainers, 2) subject and 3) time (baseline, 4 and 12 months). If necessary, analyses will be adjusted for confounders (such as age, gender, BMI, use of medication, presence of chronic diseases, physical performance, physical activity, frailty, cognition, fear of falling, walking confidence, quality of life, general health status and empowerment) and stratified for the presence of potential effect modifiers. Twisk et al. showed that it is not necessary to impute missing values using multiple imputation when performing a mixed-model analysis on longitudinal data (50).

8.2 Secondary study parameter(s)

The performance based physical functioning outcomes and other secondary outcome measures assessed with questionnaires (described in paragraph 6.1.2.) will be analyzed in a similar fashion as described above.

8.3 Other study parameters

Differences in baseline characteristics for the pre- and non-frail group will be analyzed using the student's t-test for continuous variables and Chi-squared or Fisher's exact test for categorical variables. The items in the questionnaire with respect to the process evaluation will be analyzed in a descriptive manner. The data from the focus groups will be transcribed verbatim and analyzed by two coders by using an inductive thematic approach (51). During the coding process, data will be sorted and analyzed inductively using a three-stage model of open, axial and selective coding. The primary investigator (MvG) will start with the open coding process. Subsequently, a second coder will be involved to discuss, categorize, and rename the themes into axial codes. In this stage, both researchers will explore relationships between categories and subcategories. Then, the data will be synthesized and evaluated through 'selective coding' in which the core themes will be identified. MAXQDA 20 software will be used for the qualitative analyses.

8.4 Interim analysis (if applicable)

Not applicable

9. ETHICAL CONSIDERATIONS

9.1 Regulation statement

The study will be conducted according to the principles of the Declaration of Helsinki (7th revision, October 2013) and in accordance with the Medical Research Involving Human Subjects Act (WMO) and other guidelines, regulations and Acts such as Good Clinical Practice.

9.2 Recruitment and consent

Participants will be recruited via the registered 'In Balans' trainers. In addition, participants will be recruited trough flyers (see document E3. 'Flyer 'In Balans') at annual flu shots, at supermarkets and pharmacists, and community centres where older adults meet for leisure activities. If necessary, advertisements in local papers will be used. Persons that are interested will be contacted by telephone for eligibility screening by the executive researcher (MvG). Those who are eligible will receive an extended information letter (see document E1/E2. 'Informatiebrief en toestemmingsformulier'), in which the study procedure, including randomization, will be explained. They will be asked for their informed consent for participation in the study.

9.3 Objection by minors or incapacitated subjects (if applicable)

Not applicable

9.4 Benefits and risks assessment, group relatedness

Subjects randomized to the intervention group will be invited to participate in the 'In Balans' training. The program combines educational and exercise components to raise awareness of fall risk factors, exercises to improve balance and mobility, and overall to increase self-confidence. It is assumed that participants benefit from the program since the exercises will improve their balance, mobility and self-confidence. These improvements may lead to less risk of falling, less fear of falling and therefore to a higher quality of life. The group sessions take place under supervision of a trained and certified 'In Balans' trainer that will ensure the safety of the participants. The associated risks with participation are considered negligible. For study purposes, participants will be asked to fill in a monthly fall calendar and wear an accelerometer for one week (day and night). This does not interfere with daily living and has no risks involved.

9.5 Compensation for injury

The sponsor/investigator has a liability insurance which is in accordance with CCMO format, article 7 of the WMO. This insurance provides cover for damage to research subjects through injury or death caused by the study. The insurance applies to the damage that becomes apparent during the study or within 4 years after the end of the study. This insurance covers:

- € 650.000 as maximum per claim of a research subject, with a maximum of
- € 5.000.000 for claims from one individual scientific study, limiting to
- € 7.500.000 for damage to research subjects within one year.

The insurance does not cover claims for damage to offspring due to a negative effect of participation in the medical research in the research subject or the offspring, claims for damage which could have been expected based on the nature of the medical research, and claims for damage due to not or partly following the instructions by the research subject, if the subject is capable of understanding those.

9.6 Incentives (if applicable)

No incentives will be given during the study

10. ADMINISTRATIVE ASPECTS, MONITORING AND PUBLICATION

10.1 Handling and storage of data and documents

The researcher (MvG) will give all participants a unique identification code. This identification code will consist of five digits: the first two for the 'In Balans' trainers and the last three for the participant. This code is used for all parts of the project and this code is not traceable back to the participants person. The identification number is matched with the corresponding name to inform each participant about the allocation. Only the researcher has access to the participants' details. The key to this code will be safeguarded by the researcher. Data will be stored for 15 years at the Vrije Universiteit Amsterdam. The informed consents will be the only documents with both the name and the identification code on it. These forms will primary be stored in a locked office and as electronic information on a password-protected server at VU University. This server is only accessible by the researcher and supervisor. Demographic data and data collected during the study will be collected in a password-protected

internet-based database. Data will be handled confidentially. Anyone with access to the data, including the investigator, is subject to professional secrecy during and after the project. Only members of the research team, monitors and supervisory authorities have insight into the data. The handling of personal data will comply with the EU General Data Protection Regulation and the Dutch Act on Implementation of the General Data Protection Regulation (Wet bescherming persoonsgegevens and AVG).

10.2 Monitoring and Quality Assurance

Since the risk of damage is considered negligible no external independent monitor will be installed. Instead, an internal monitor with peers will be installed who perform monitoring visits to all participating sites and review the adherence to the study protocol and verify source documents, such as informed consent.

10.3 Amendments

Amendments are changes made to the research after a favourable opinion by the accredited METC has been given. All amendments will be notified to the METC that gave a favourable opinion.

10.4 Annual progress report

The sponsor/investigator will submit a summary of the progress of the trial to the accredited METC once a year. Information will be provided on the date of inclusion of the first subject, numbers of subjects included and numbers of subjects that have completed the trial, serious adverse events/ serious adverse reactions, other problems, and amendments.

10.5 Temporary halt and (prematurely) end of study report

The investigator/sponsor will notify the accredited METC of the end of the study within a period of 8 weeks. The end of the study is defined as the last subjects' last visit. The sponsor will notify the METC immediately of a temporary halt of the study, including the reason of such an action. In case the study is ended prematurely, the sponsor will notify the accredited METC within 15 days, including the reasons for the premature termination. Within one year after the end of the study, the investigator/sponsor will submit a final study report with the results of the study, including any publications/abstracts of the study, to the accredited METC.

10.6 Public disclosure and publication policy

Notification and registration of this randomized controlled trial in the Trial Registry will take place after the approval of METC and before the first subject is recruited. This research proposal is part of a PhD program. Results of the study will be, in accordance with the CCMO statement on publication policy, published in peer-reviewed international medical scientific journals and also in non-peer reviewed magazines for health professionals.

11. REFERENCES

1. Faber MJ, Bosscher RJ, Paw MJCA, van Wieringen PC. Effects of exercise programs on falls and mobility in frail and pre-frail older adults: a multicenter randomized controlled trial. Archives of physical medicine and rehabilitation. 2006;87(7):885-96.
2. CBS. Bevolking; kerncijfers 2019 [cited 2020 13/09]. Available from: https://opendata.cbs.nl/statline/#/CBS/nl/dataset/37296ned/table?ts=1565098840640.
3. van der Does HB, A; Panneman, A. . Privé- valongevallen bij ouderen: Cijfers valongevallen in de privésfeer 2018. Amsterdam: VeiligheidNL; 2019.
4. Sherrington C, Fairhall NJ, Wallbank GK, Tiedemann A, Michaleff ZA, Howard K, et al. Exercise for preventing falls in older people living in the community. Cochrane Database Syst Rev. 2019;1:CD012424.
5. Nyman SR, Victor CR. Older people's participation in and engagement with falls prevention interventions in community settings: an augment to the Cochrane systematic review. Age and ageing. 2012;41(1):16-23.
6. Pijnappels MvS, K.S.; Sluijter, A.M.C.;Meskers, C.G.M.; Maier A.B. Beweegstimulatie en valpreventie: overzicht van effectieve interventies voor zelfstandig wonende ouderen. Nederlands Tijdschrift voor Geriatriefysiotherapie. 2018:13.
7. VeiligheidNL. Keuzehulp beweegprogramma's Valpreventie 65+. 2019.
8. Zijlstra GA, van Haastregt JC, Ambergen T, van Rossum E, van Eijk JT, Tennstedt SL, et al. Effects of a multicomponent cognitive behavioral group intervention on fear of falling and activity avoidance in community-dwelling older adults: results of a randomized controlled trial. J Am Geriatr Soc. 2009;57(11):2020-8.
9. Thomas S, Mackintosh S, Halbert J. Does the 'Otago exercise programme' reduce mortality and falls in older adults?: a systematic review and meta-analysis. Age Ageing. 2010;39(6):681-7.
10. Weerdesteyn V, Smulders E, Rijken H, Duysens J. Preserved effectiveness of a falls prevention exercise program after implementation in daily clinical practice. J Am Geriatr Soc. 2009;57(11):2162-4.
11. Weerdesteyn V, Rijken H, Geurts AC, Smits-Engelsman BC, Mulder T, Duysens J. A five-week exercise program can reduce falls and improve obstacle avoidance in the elderly. Gerontology. 2006;52(3):131-41.
12. Bemer EB, M. . In Balans: doelgroep in haar context Social Marketing. Amsterdam: TNS-NIPO; 2014.
13. ProjectTOM. Thuis Onbezorgd Mobiel (TOM). 2019.
14. Jansen-Kosterink S, van Velsen L, Frazer S, Dekker-van Weering M, O'Caoimh R, Vollenbroek-Hutten M. Identification of community-dwelling older adults at risk of frailty using the PERSSILAA screening pathway: a methodological guide and results of a large-scale deployment in the Netherlands. BMC Public Health. 2019;19(1):504.
15. Peeters G, van Schoor NM, Lips P. Fall risk: the clinical relevance of falls and how to integrate fall risk with fracture risk. Best Pract Res Clin Rheumatol. 2009;23(6):797-804.
16. VeiligheidNl. Valanalyse voor de eerstelijnszorg 2020 [cited 2020 13/10]. Available from: https://www.veiligheid.nl/valpreventie/interventies/screening/valanalyse.
17. Steverink NS, J.; Schuurmans, H.; van Lis, M. Measuring frailty: developing and testing of the Groningen Frailty Indicator (GFI). Gerontologist. 2001;41.
18. Fried LP, Tangen CM, Walston J, Newman AB, Hirsch C, Gottdiener J, et al. Frailty in older adults: evidence for a phenotype. J Gerontol A Biol Sci Med Sci. 2001;56(3):M146-56.
19. Sherrington C, Fairhall NJ, Wallbank GK, Tiedemann A, Michaleff ZA, Howard K, et al. Exercise for preventing falls in older people living in the community. Cochrane database of systematic reviews. 2019(1).
20. VeiligheidNL. Scholing In Balans 2020 [cited 2020 29/09]. Available from: https://www.veiligheid.nl/valpreventie/trainingen/scholing-in-balans.
21. Weggemans RM, Backx FJG, Borghouts L, Chinapaw M, Hopman MTE, Koster A, et al. The 2017 Dutch Physical Activity Guidelines. Int J Behav Nutr Phys Act. 2018;15(1):58.
22. Lamb SE, Jorstad-Stein EC, Hauer K, Becker C, Prevention of Falls Network E, Outcomes Consensus G. Development of a common outcome data set for fall injury prevention trials: the Prevention of Falls Network Europe consensus. J Am Geriatr Soc. 2005;53(9):1618-22.
23. EQ-5D. EQ-5D Instruments 2020 [cited 2020 29/09]. Available from: https://euroqol.org/.
24. Rand S, Caiels J, Collins G, Forder J. Developing a proxy version of the Adult social care outcome toolkit (ASCOT). Health and Quality of Life Outcomes. 2017;15(1):108.
25. Bouwmans CH-vR, L.; Koopmanschap, M.; Krol, M.; Severens, H.; Brouwer, W. Medical Consumption Questionnaire. Rotterdam: Institute for Medical Technology Assessment 2013.
26. Bouwmans CH-vR, L.; Koopmanschap, M.; Krol, M.; Severens,H.; dr. Brouwer, W. Productivity Cost Questionnaire. Rotterdam Institute for Medical Technology Assessment; 2013.
27. Tinetti ME. Performance-oriented assessment of mobility problems in elderly patients. Journal of the American Geriatrics Society. 1986.
28. Nelson J. The Effectiveness of the" Stepping On" Program for Reducing the Incidence of Falls in the Elderly: Measured by Four Stage Balance Test. 2016.

29. Mathias S, Nayak U, Isaacs B. Balance in elderly patients: the" get-up and go" test. Archives of physical medicine and rehabilitation. 1986;67(6):387-9.
30. Rantanen T, Guralnik JM, Foley D, Masaki K, Leveille S, Curb JD, et al. Midlife hand grip strength as a predictor of old age disability. Jama. 1999;281(6):558-60.
31. Jones CJ, Rikli RE, Beam WC. A 30-s chair-stand test as a measure of lower body strength in community-residing older adults. Research quarterly for exercise and sport. 1999;70(2):113-9.
32. Guralnik JM, Simonsick EM, Ferrucci L, Glynn RJ, Berkman LF, Blazer DG, et al. A short physical performance battery assessing lower extremity function: association with self-reported disability and prediction of mortality and nursing home admission. Journal of gerontology. 1994;49(2):M85-M94.
33. Collen FM, Wade DT, Bradshaw CM. Mobility after stroke: reliability of measures of impairment and disability. International disability studies. 1990;12(1):6-9.
34. Rikli RE, Jones CJ. Development and validation of a functional fitness test for community-residing older adults. Journal of aging and physical activity. 1999;7(2):129-61.
35. Tromp A, Pluijm S, Smit J, Deeg D, Bouter L, Lips P. Fall-risk screening test: a prospective study on predictors for falls in community-dwelling elderly. Journal of clinical epidemiology. 2001;54(8):837-44.
36. Van Schooten KS, Van Dieen JH, Pijnappels M, Maier AB, van't Hul AJ, Niessen M, et al. The association between age and accelerometry-derived types of habitual daily activity: an observational study over the adult life span in the Netherlands. BMC public health. 2018;18(1):824.
37. Van Schooten KS, Pijnappels M, Rispens SM, Elders PJ, Lips P, Daffertshofer A, et al. Daily-life gait quality as predictor of falls in older people: a 1-year prospective cohort study. PLoS one. 2016;11(7):e0158623.
38. Ware Jr JE. SF-36 health survey. 1999.
39. Flinterman F, Lauriks S, de Wit M, Cremer S, Fassaert T, Verhoeff A, et al. Mijn Positieve Gezondheid en de Zelfredzaamheid-Matrix. TSG-Tijdschrift voor gezondheidswetenschappen. 2019;97(7-8):160-4.
40. Pearlin LI, Schooler C. The structure of coping. Journal of health and social behavior. 1978:2-21.
41. Luszczynska A, Scholz U, Schwarzer R. The general self-efficacy scale: multicultural validation studies. The Journal of psychology. 2005;139(5):439-57.
42. Yardley L, Beyer N, Hauer K, Kempen G, Piot-Ziegler C, Todd C. Development and initial validation of the Falls Efficacy Scale-International (FES-I). Age and ageing. 2005;34(6):614-9.
43. Newell AM, VanSwearingen JM, Hile E, Brach JS. The modified gait efficacy scale: establishing the psychometric properties in older adults. Physical therapy. 2012;92(2):318-28.
44. Association WSH. Partnership for patients 2018 [cited 2020 29/09]. Available from: https://www.wsha.org/partnershipforpatients.cfm.
45. Steverink N. Measuring frailty: developing and testing the GFI (Groningen Frailty Indicator). The Gerontologist. 2001;41:236.
46. van Buuren S, Boshuizen HC, Knook DL. Multiple imputation of missing blood pressure covariates in survival analysis. Stat Med. 1999;18(6):681-94.
47. Fenwick E, O'Brien BJ, Briggs A. Cost-effectiveness acceptability curves--facts, fallacies and frequently asked questions. Health Econ. 2004;13(5):405-15.
48. ZorginstituutNederland;. Richtlijn voor het uitvoeren van economische evaluaties in de gezondheidszorg. Diemen: Zorginstituut Nederland; 2016.
49. Sullivan SD, Mauskopf JA, Augustovski F, Jaime Caro J, Lee KM, Minchin M, et al. Budget impact analysis-principles of good practice: report of the ISPOR 2012 Budget Impact Analysis Good Practice II Task Force. Value Health. 2014;17(1):5-14.
50. Twisk J, de Boer M, de Vente W, Heymans M. Multiple imputation of missing values was not necessary before performing a longitudinal mixed-model analysis. J Clin Epidemiol. 2013;66(9):1022-8.
51. Straus ALC, J. Basic of Qualitative Research: Grounded Theory Procedures and Techniques. second ed. London: SAGE Publications; 1990.

Attachment 1: Overview of the measurements

Activities	Assessment	Baseline	4 months	8 months	12 months
**Screening by telephone**	x				
**Informed consent**	x				
**Randomization**	x				
**Assessment and condition**<br>- Personal characteristics<br>- Several physical activity, strength and balance measurements<br>- Questionnaires about physical activity, health status and cognition	x				
**Measurements for exercise performance**<br>- Balance<br>- Strength<br>- Mobility		x	x		x
**Questionnaires**<br>- Demographic characteristics (for example age, gender, living situation, etc.)<br>- Fall risk<br>- Fear of falling<br>- Quality of life<br>- Health status<br>- Daily exercise		x	x		x
**Questionnaires**<br>- Use of healthcare<br>- Loss of productivity			x	x	x
**Measuring daily activity** with a move monitor for 7 days		x	x		x
