# Supplementary material for: The cost-effectiveness of the Dutch In Balance fall prevention intervention compared to exercise recommendations among community-dwelling older adults with an increased risk of falls: A randomized controlled trial
Source: PLoS One. 2025 Dec 30;20(12):e0339497. doi: 10.1371/journal.pone.0339497 (PMC12752955; doi:10.1371/journal.pone.0339497)
Supplement: Attachment — Submitted filename: Response to reviewers.docx [file pone.0339497.s011.docx]

Dear dr. Koleva-Kolarova,

Thank you for giving us the opportunity to revise our manuscript entitled ‘*The cost-effectiveness of the Dutch In Balance fall prevention intervention compared to exercise recommendations among community-dwelling older adults with an increased risk of falls: a randomized controlled trial’* and the willingness to consider our manuscript for publication in PLOS ONE*.* We appreciate the fast review process concerning our manuscript. Please find our responses to the reviewers' comments below. The phrases in italics are changed or added in the revised manuscript. We want to thank the reviewers for their comments which improved our manuscript. We hope that the revised manuscript is acceptable for publication.

**Journal requirements:**

Comment 1: Please ensure that your manuscript meets PLOS ONE's style requirements, including those for file naming. The PLOS ONE style templates can be found at

https://journals.plos.org/plosone/s/file?id=wjVg/PLOSOne_formatting_sample_main_body.pdf and

https://journals.plos.org/plosone/s/file?id=ba62/PLOSOne_formatting_sample_title_authors_affiliations.pdf.

*Response and action: We have reviewed the PLOS ONE style requirements and updated the manuscript and file naming accordingly, using the provided templates.*

Comment 2: Thank you for stating the following in the Competing Interests section:

“The first authors (JD and MvG) are early-stage investigators employed by the Vrije Universiteit Amsterdam. MvG coordinated the data collection and JD performed the analysis of the data. JD, MvG, PV, DB, BV, MP and JB have no competing interests. One author (BO) is employed by VeiligheidNL, which is the institute that owns the In Balance intervention. VeiligheidNL helped in designing the study, but was not involved in the data analysis and did not have an influence on the results in any way. No funding has been received from a commercial organization.”

We note that one or more of the authors are employed by a commercial company: name of commercial company.

Comment 2a: Please provide an amended Funding Statement declaring this commercial affiliation, as well as a statement regarding the Role of Funders in your study. If the funding organization did not play a role in the study design, data collection and analysis, decision to publish, or preparation of the manuscript and only provided financial support in the form of authors' salaries and/or research materials, please review your statements relating to the author contributions, and ensure you have specifically and accurately indicated the role(s) that these authors had in your study. You can update author roles in the Author Contributions section of the online submission form.

Please also include the following statement within your amended Funding Statement.

“The funder provided support in the form of salaries for authors [insert relevant initials], but did not have any additional role in the study design, data collection and analysis, decision to publish, or preparation of the manuscript. The specific roles of these authors are articulated in the ‘author contributions’ section.”

If your commercial affiliation did play a role in your study, please state and explain this role within your updated Funding Statement.

*Response and action: We would like to point out that VeiligheidNL is not a commercial company, but a non-profit knowledge institute with the status of a Public Benefit Organisation. They are member of Eurosafe, the European Association of Injury Prevention and Safety Promotion and aim to reduce the number of accidents and injuries in the Netherlands. As such, fall prevention is one of their key themes for injury prevention and public safety (*[*https://www.veiligheid.nl/about-us*](https://www.veiligheid.nl/about-us)*).*

*To clarify, we adapted the Funding Statement to: ‘This research was funded by a research grant (#555002018) from the Netherlands Organisation for Health Research and Development (ZonMw). The funder provided support in the form of salaries for authors MvG, PV, DB, BV, MP and JB and study costs, but did not have any additional role in the study design, data collection and analysis, decision to publish, or preparation of the manuscript. The specific roles of these authors are articulated in the ‘author contributions’ section. One author (BO) is employed by the VeiligheidNL foundation, which is a non-profit public benefit organisation and a knowledge institute for injury prevention and public safety that owns the In Balance intervention. VeiligheidNL participated in designing the study, but did not have any additional role in the data collection and analysis, decision to publish, or preparation of the manuscript. No funding has been received from any commercial organization.’*

Comment 2b: Please also provide an updated Competing Interests Statement declaring this commercial affiliation along with any other relevant declarations relating to employment, consultancy, patents, products in development, or marketed products, etc.

Within your Competing Interests Statement, please confirm that this commercial affiliation does not alter your adherence to all PLOS ONE policies on sharing data and materials by including the following statement: "This does not alter our adherence to PLOS ONE policies on sharing data and materials.” (as detailed online in our guide for authors http://journals.plos.org/plosone/s/competing-interests) . If this adherence statement is not accurate and there are restrictions on sharing of data and/or materials, please state these. Please note that we cannot proceed with consideration of your article until this information has been declared.

Please include both an updated Funding Statement and Competing Interests Statement in your cover letter. We will change the online submission form on your behalf.

*Response and action: We adapted the Competing Interests Statement to: ‘The first authors (JD and MvG) are early-stage investigators employed by the Vrije Universiteit Amsterdam. MvG coordinated the data collection and JD performed the analysis of the data. JD, MvG, PV, DB, BV, MP and JB have no competing interests. One author (BO) is employed by the VeiligheidNL foundation, which is a non-profit public benefit organisation and a knowledge institute for injury prevention and public safety that owns the In Balance intervention. VeiligheidNL participated in designing the study, but did not have any additional role in the data collection and analysis, decision to publish, or preparation of the manuscript. This does not alter our adherence to PLOS ONE policies on sharing data and materials.’*. *The updated Funding Statement and Competing Interests Statement are included in our cover letter.*

Comment 3: In the online submission form, you indicated that [Insert text from online submission form here].

All PLOS journals now require all data underlying the findings described in their manuscript to be freely available to other researchers, either 1. In a public repository, 2. Within the manuscript itself, or 3. Uploaded as supplementary information.

This policy applies to all data except where public deposition would breach compliance with the protocol approved by your research ethics board. If your data cannot be made publicly available for ethical or legal reasons (e.g., public availability would compromise patient privacy), please explain your reasons on resubmission and your exemption request will be escalated for approval.

*Response and action: This study involves human participant data that cannot be publicly shared due to ethical and privacy considerations in accordance with the protocol approved by the research ethics board. Therefore, the full dataset is not publicly available but can be shared upon reasonable request. Data requests will be assessed on a case-by-case basis to ensure that participant confidentiality is safeguarded. Requests can be directed to* [*m.pijnappels@vu.nl*](mailto:m.pijnappels@vu.nl)*. Relevant data supporting the findings are included in the manuscript.*

Comment 4: Thank you for stating the following in the Acknowledgments Section of your manuscript:

“We want to thank all participants and therapists for participating in this study. Moreover, this research was funded by a research grant (#555002018) from the Netherlands Organisation for Health Research and Development (ZonMw). The funders had no role in study design, data collection and analysis, decision to publish, or preparation of the manuscript. We also want to thank VeiligheidNL, in particular Sanne Frazer, for their help with designing this study. “

We note that you have provided additional information within the Acknowledgements Section that is not currently declared in your Funding Statement. Please note that funding information should not appear in the Acknowledgments section or other areas of your manuscript. We will only publish funding information present in the Funding Statement section of the online submission form.

Please remove any funding-related text from the manuscript and let us know how you would like to update your Funding Statement. Currently, your Funding Statement reads as follows:

“This research was funded by a research grant (#555002018) from the Netherlands Organisation for Health Research and Development (ZonMw). The funders had no role in study design, data collection and analysis, decision to publish, or preparation of the manuscript.”

Please include your amended statements within your cover letter; we will change the online submission form on your behalf.

*Response and action: We removed the funding information from the Acknowledgements Section, which now reads: ‘We want to thank all participants and therapists for participating in this study. We also want to thank VeiligheidNL, in particular Sanne Frazer, for their help with designing this study.’. For the adapted Funding Statement, please refer to our response to comment 2a.*

Comment 5: Your ethics statement should only appear in the Methods section of your manuscript. If your ethics statement is written in any section besides the Methods, please delete it from any other section.

*Response and action: Thank you for the clarification. We have revised the manuscript to ensure that the ethics statement now appears exclusively in the Methods section, and have removed it from all other sections.*

Comment 6: Please include captions for your Supporting Information files at the end of your manuscript, and update any in-text citations to match accordingly. Please see our Supporting Information guidelines for more information: <http://journals.plos.org/plosone/s/supporting-information>.

*Response and action: We have now added captions for all Supporting Information files at the end of the manuscript, in accordance with PLOS ONE’s guidelines. Additionally, we have reviewed and updated all in-text citations to ensure they correctly correspond to the revised Supporting Information section.*

**Additional editor comments:**

General comment: Dear Authors, Thank you for submitting your manuscript to Plos One. I would like to state that this is a well-conducted economic analysis and a well-written article which I enjoyed reading. I have now reached a decision which is Major Revision. In addition to the comments from the reviewers, please see below my comments:

*Response: Thank you for your positive feedback and for the opportunity to revise our manuscript. We have carefully addressed your comments as well as those from the reviewers.*

Comment 1: Could the authors please justify not including non-Dutch speakers in the study?

*Response and action: Non-Dutch speakers were not included in the study because In Balance is a Dutch program, delivered entirely in Dutch and accompanied by Dutch-language materials.*

*We included this in the Methods section of the manuscript on page 7 as follows: ‘Other exclusion criteria were the inability to walk 100 meters independently or to perform activities of daily living without assistance, cognitive impairment defined as a score lower of 18 or lower on the Mini-Mental State Examination* [1]*, self-reported contraindications (i.e., cardiovascular, neurological and orthopedic problems), participation in a fall prevention intervention within the last 6 months, or insufficient proficiency in Dutch, as the In Balance intervention and all materials were only available in Dutch.’*

Comment 2: Similarly to the comment from another reviewer, could the authors please clarify why they considered productivity losses in a population group of elderly and potentially retired participants? Could the authors please elaborate if excluding productivity losses would have an impact on cost-effectiveness results?

*Response and action: Although the majority of participants were retired since the official retirement age in the Netherlands is 67 years, our inclusion criterion was being aged 65 years and older, and 14 participants in our study were still employed. Additionally, 22 participants reported being unable to perform unpaid work due to health complaints. To ensure a broad, comprehensive societal assessment of costs, we chose to include productivity losses, both from paid and unpaid work, in our analysis. From a societal perspective, these costs are relevant and should be taken into account. Moreover, our data confirm that some participants were still working at the time of the study. Exclusion of these costs results in slightly lower total cost estimates, but does not change the overall conclusions regarding the cost-effectiveness of the In Balance program as shown by our sensitivity analysis from the healthcare perspective.*

*We included this in the Methods section of the manuscript on page 8 as follows: ‘Although most participants were retired, 14 were still employed and 22 reported being unable to perform unpaid work due to health issues. Given the Dutch recommendation to take a broad societal perspective, productivity losses, both paid and unpaid, were included to provide a comprehensive estimate of costs [2].’*

General comment: Thank you. Good luck with revising the article.

Response: Thank you. We did our best to address all comments thoroughly in the revision.

**Reviewer 1:**

General comment: This manuscript describes a well-conducted economic evaluation of the In Balance intervention compared with physical activity recommendations in non-frail and pre-frail older adults with a high fall risk. The manuscript reads well and the provided information is clear. Findings support the conclusion. Some clarifications would be helpful on methodological choices that were made.

Response: We thank the reviewer for the positive and encouraging feedback. We appreciate your recognition that the manuscript reads well, the information provided is clear, and that the findings support the conclusion. In response to your suggestion, we have added clarifications on specific methodological choices. We hope these additions improve the transparency of our methods.

Comment 1: Introduction (p. 4, line 65): You say that In Balance is a widely implemented intervention. Is it widely implemented in the Netherlands or Europe or the world? In line 74 you say that it is important to evaluate the cost-effectiveness to inform policy makers to optimally allocate resources or to arrange reimbursement. Given that it is already implemented, how is the intervention financed now? Are there concerns regarding implementation (e.g. are the right people reached)? In other words, why is it important to do the cost-effectiveness evaluation now that the intervention is already implemented?

*Response and action: Thank you for this thoughtful comment. We have clarified in the manuscript that In Balance is a widely implemented intervention in the Netherlands (page 4, line 68). At present, the national government provides funding to municipalities, which are responsible for implementing fall prevention programs such as In Balance at the local level. Health insurers only reimburse the program when there is an underlying medical condition. In some cases, the program is (partly) reimbursed through supplementary insurance. Otherwise, participants in most municipalities pay a personal contribution.*

*We agree that it is important to justify why a cost-effectiveness evaluation is needed given that the program is already in use. As such, we have elaborated this in the Introduction section as follows: ’The cost-effectiveness of In Balance has not been researched before, while this information is essential to assess whether the program provides value for money. Demonstrating cost-effectiveness could support further implementation and strengthen the case for broader reimbursement by health insurers, and may also support decision-making when choosing between different fall prevention programs.’*

Comment 2: Introduction (p. 5, line 85): why was physical activity recommendations (line 85) or exercise recommendations (line 87) chosen as the control condition? Can this be considered usual care or an active (or passive?) control condition?

*Response and action: For consistency, we have replaced ‘exercise recommendations’ with ‘physical activity recommendations’ in line 93 in the Introduction section. We consider this control condition to reflect usual care. We chose this approach to provide control group participants with some minimal information, aiming to maintain their engagement in the study. However, we did not expect them to take further action based on the flyer. Therefore, we regard this as a usual care condition rather than an active or passive control.*

*We added this to the Introduction section as follows: ‘Physical activity recommendations were used as a usual care condition to maintain participant engagement without expecting substantial behavioral change.’.*

Comment 3: Methods – Participants (p. 6, line 109): Why were frail participants excluded?

*Response and action: Frail participants were excluded because a previous study showed that participation in the In Balance intervention was associated with an increased risk of falling among frail older adults (hazard ratio = 2.95; 95% CI, 1.64–5.32) [3]. To ensure participant safety and avoid potential harm, we decided not to include frail individuals in this study.*

*We included the following in the Methods section on page 5, line 120-122: ‘Participants who met three criteria or more were classified as frail and therefore excluded from the study, since previous research found an increased fall risk in frail older adults after participating in In Balance* [3]*.’*

Comment 4: Methods – Intervention and control group (p. 7, line 131): Could you please describe briefly what these physical activity recommendations were?

*Response and action: We added in the Methods section at page 7, line 148-153: ‘The control group received a flyer containing advice on physical activity levels, strength and balance for older adults. For example, a minimal amount of 150 minutes of moderate to vigorous physical activity per week was recommended, distributed over several days. Also, muscle and bone strengthening activities such as walking stairs and balance exercises at least twice per week are advised. Moreover, the health benefits associated with physical activity are explained. These recommendations are according to the physical activity guidelines of the World Health Organization [4].’*

Comment 5: Methods - Costs (p. 7/8): Were costs of the intervention itself measured and if so, how?

*Response and action: The costs of the In Balance intervention were estimated according to a template of VeiligheidNL, and divided among the maximum number of 12 participants per training group, see S1 for an overview of this calculation. This is described in the Methods section on page 9, lines 199–202: ‘The costs of the In Balance intervention were estimated according to a template of VeiligheidNL, and divided among the maximum number of 12 participants per training group, see S1 for an overview of this calculation.’.*

Comment 6: Methods – Costs (p. 8, line 139): Given the inclusion criterion of being aged 65 years and over, and the retirement age being 67, I wonder how relevant it was to include costs of lost productivity (IPCQ). With the average age being 75 years, how many participants were in paid work? Unpaid work is probably more important in this age-range. The types of activities people do and the hours per week varies a lot. How reliable/realistic is it to use one shadow price for all these different activities?

*Response and action: As also described in our response to comment 2 of the additional editor comments, although the majority of participants were retired since the official retirement age in the Netherlands is 67 years, our inclusion criterion was being aged 65 years and older, and 14 participants in our study were still employed. Additionally, 22 participants reported being unable to perform unpaid work due to health complaints. To ensure a broad, comprehensive assessment of costs, we chose to include productivity losses, both from paid and unpaid work, in our analysis. From a societal perspective, these costs are relevant and should be taken into account. Moreover, our data confirm that some participants were still working at the time of the study. Exclusion of these costs of productivity losses results in slightly lower total cost estimates, but does not change the overall conclusions regarding the cost-effectiveness of the In Balance program as shown by our sensitivity analysis from the healthcare perspective.*

*We included this in the Methods section of the manuscript on page 8 as follows: ‘Although most participants were retired, 14 were still employed and 22 reported being unable to perform unpaid work due to health issues. Given the Dutch recommendation to take a broad societal perspective, productivity losses, both paid and unpaid, were included to provide a comprehensive estimate of costs [2]’.*

*We acknowledge that the type and amount of unpaid work vary substantially between individuals. However, to allow comparability across studies and follow national guidelines for economic evaluations, we used a standardized shadow price as recommended in the Dutch costing manual [2].*

Comment 7: Methods – Sample size (p. 0): An expected effect size of 50% seems overly optimistic given effects founds in previous falls prevention trials. What was this expectation based on?

*Response and action: We acknowledge that an expected effect size of 50% was an ambitious assumption used in the sample size calculation. The rationale behind this was our expectation of a substantial effect to ensure clinical relevance. While we recognize this was a bold assumption, our intention was to detect a meaningful reduction in fall incidence that could justify large-scale implementation of the program. Besides, this estimate was based on the study by Faber et al., which previously evaluated the effects of the In Balance program and also used an effect size of 50% reduction for their sample size calculation [3].*

*We added in the Methods section at page 10, lines 227-229: ‘The expected 50% reduction in falls was chosen to reflect a clinically relevant effect and was also based on a study of Faber and colleagues, who used a similar estimate [3].’.*

Comment 8: Methods - Imputation (p. 10): What was the proportion and pattern of missing values? Later I noted that this information was provided in the Results on page 12. Perhaps consider moving this information to the methods, so that the reader understand methodological choices that were made early on.

*Response and action: We moved the following paragraph from the Results section to the Methods section at page 10 and 11, lines 243-247: ‘The rate of missing data was 10.4% for fall diaries and follow-up calls, and 16.5% for cost and quality of life questionnaires in the intervention group. In the control group, the percentages of missing data were 15.0% and 26.1%, respectively. Participants with complete data had statistically significantly higher Mini-Mental State Examination scores (indicating better cognitive functioning) and a lower fall history at baseline.’.*

Comment 9: Results – Table 1: How were the descriptive variables measured, particularly physical activity?

*Response and action: We added a heading ‘Descriptive variables’ to the Methods section on page 11, lines 252-256, in which we describe how the descriptive variables were measured, particularly physical activity: ‘All descriptive variables were collected using questionnaires. Physical activity, however, was assessed with an inertial sensor (DynaPort MoveMonitor Plus, McRoberts BV, The Netherlands)* [5, 6]*. The sensor was worn on the lower back for seven consecutive days, preferably day and night except during water activities, and was returned by mail afterwards.’*

Comment 10: Results – Adverse events (p. 19): Please describe how adverse events were measured in the methods.

*Response and action: We added a heading ‘Adverse events’ to the Methods section on page 12, lines 294-299, in which we describe how adverse events were measured: ‘Adverse events were defined as any undesirable experience occurring to a participant during the study, whether or not considered related to the In Balance intervention or the trial procedure. All adverse events reported spontaneously by the participant or observed by the investigator or staff were recorded. Occurrence of adverse events was assessed during the monthly telephone calls and for the intervention group during the In Balance intervention by the therapist.’.*

Comment 11: Discussion (p. 20, line 349): You describe here that your results align with similar recent international studies, but how does it compare with other Dutch studies, for example:

o Hendriks et al. Cost-effectiveness of a multidisciplinary fall prevention program in community-dwelling elderly people: a randomized controlled trial (ISRCTN 64716113). Int J Technol Assess Health Care. 2008 Spring;24(2):193-202. doi: 10.1017/S0266462308080276. PMID: 18400123.

o Peeters et al. Multifactorial evaluation and treatment of persons with a high risk of recurrent falling was not cost-effective. Osteoporos Int. 2011 Jul;22(7):2187-96. doi: 10.1007/s00198-010-1438-4.

As the health care system and access to services has a great influence on costs, comparison of results with studies within the same/similar health care system may be more relevant than comparison with studies in different countries/health care systems.

*Response and action: We included the two above mentioned studies in the Discussion section and compared their results with the results of our study (lines 434-444): ‘Other Dutch cost-effectiveness evaluations by Hendriks and colleagues (2008) and Peeters and colleagues (2011) of (multidisciplinary) fall prevention interventions also found no statistically significant differences in falls, costs, and quality of life, similar to our findings [7, 8]. However, both studies concluded that their intervention were not cost-effective, whereas In Balance showed a 98% probability of being cost-effective due to lower costs in the In Balance group [7, 8]. This difference may relate to the inclusion of participants and nature of the intervention: Hendriks et al. evaluated medical and occupational assessments (e.g. assessments of general physical examination and daily functioning) [7], and Peeters et al. focused on multifactorial evaluation and treatment of persons with a high risk of recurrent falls [8], while In Balance combines exercise and education, which is an approach more generally proven to reduce falls [9].’.*

Comment 12: Discussion (p. 21, line 378): If I interpret this sentence correctly, then you describe here that the sample was representative of the population that typically receives the In Balance intervention. The level of education in this sample was quite high. Does this reflect the general population that receives In Balance? Might this suggest that extra efforts are needed to reach groups with lower levels of education?

*Response and action: We acknowledge that the level of education in our study population was relatively high. This is indeed representative of the population that typically participates in the In Balance intervention, which often includes higher educated individuals, predominantly women, and mostly people without a migration background. We agree with your suggestion that extra efforts are needed to reach a population that better reflects society as a whole, including people with lower levels of education and those with a migration background.*

*We included this in the discussion section on page 22 in lines 421-423 as follows: ‘This highlights the need for additional efforts to also include older adults with for example lower educational levels and migration backgrounds in the In Balance intervention to ensure broader societal representation.’.*

Comment 13: Discussion (p. 21, lines 470-472): “Moreover, although we used an optimal number of …. of the In Balance intervention.” Apologies but I do not understand this sentence or what is meant here. Which groups are you talking about here? Weren’t there 131 and 133 participants in the intervention and control groups, respectively? Why 12 per group? Can you please rephrase to clarify the point you are making here?

*Response and action: Our apologies for the confusion. With this sentence, we aimed to explain that the In Balance intervention is delivered in a group setting, and for the calculation of intervention costs per participant, we assumed an optimal group size of 12 participants. However, in our study, and in practice, several groups consisted of fewer than 12 participants, which can lead to higher per-person costs.*

*We adapted the sentence in the Discussion section on page 22-23, lines 486-489 to: ‘Moreover, the In Balance intervention is delivered in a group setting, and intervention costs per participant were calculated based on an optimal group size of 12 participants per group. However, as several groups were smaller in practice, the actual costs per participant may have been higher.’.*

**Reviewer 2:**

General comment: Dear Editor, I write to submit my comments on the manuscript titled “The cost-effectiveness of the Dutch In Balance fall prevention intervention compared to exercise recommendations among community-dwelling older adults with an increased risk of falls: a randomized controlled trials”. The study, via a randomized controlled trial, evaluated the cost-effectiveness of the In Balance fall prevention intervention for community-dwelling older adults with an increased risk of falls compared to general physical activity recommendations (control) from a societal perspective. Here are my comments.

Response: *We thank you for your detailed and thorough comments. We believe your suggestions have strengthened the quality and clarity of the work. We hope that our clarifications and additional analyses adequately address your concerns and contribute to improving the manuscript further.*

Comment 1: The outcome measures are the number of falls, fall-related injuries, and Quality-Adjusted Life Years (QALYs) based on the EuroQol Five-level questionnaire (EQ-5D-5L) and the Adult Social Care Outcomes Toolkit (ASCOT). 1) Clarification: The authors indicated that the control group received written general recommendations on physical activity via a flyer, in accordance with the World Health Organization's physical activity guidelines and the intervention group received the “In Balance”. Is it the case that the intervention group actually received the physical activity in accordance with the World Health Organization's physical activity guidelines plus the “In Balance,” OR they only received just the Inbalance? That is control=A and Intervention=A+B. Kindly provide clarity on that. This is also important because the intervention group may be involved in physical activities similar to those described in the WHO physical activity guidelines.

*Response and action: The intervention group participated exclusively in the In Balance program and did not receive the World Health Organization’s physical activity guidelines in addition to the In Balance program. While the In Balance program includes educational components that align with the WHO recommendations, these guidelines were not explicitly provided as separate written materials.*

*We included the sentence on page 7 (line 144-146): ‘Participants in the intervention group received only the In Balance program as part of the study protocol and were not additionally provided with other materials on physical activity.’.*

Comment 2: In line 133, the authors indicated outcome measure and mentioned cost but also said also said in line 159 that the primary outcome measures in this trial were the number of falls and the number of injuries due to a fall

*Response and action: We agree that this is somewhat unclear. The number of falls and injuries are the primary outcome of the randomized controlled trail, and the QALYs the primary outcome of the economic evaluation. In line with this the cost should be reported following the description of the effects.*

*We have moved the paragraph on costs to follow directly after the paragraph on effects in the outcome section of the method.*

Comment 3: Authors indicated that multiple imputation by chained equations were used but authors did not provide any information on the missing mechanism assumption (missing at random, missing completely at random and missing not at random) and justification as to the choice of a selected missing mechanism

*Response and action: We agree that the assumption regarding the missing data mechanism should have been stated more explicitly. The pattern of missing data showed that participants with complete data had statistically significantly higher Mini-Mental State Examination (MMSE) scores and a lower fall history at baseline, compared with participants with missing data. These observed associations indicate that the missingness is related to baseline characteristics, and therefore, the assumption of Missing Completely at Random (MCAR) is not supported. We conducted the subsequent analyses under the Missing at Random (MAR) assumption. To strengthen the plausibility of this assumption, both MMSE scores and fall history were included as predictors in the multiple imputation model [10].*

*We included the following sentence in the data imputation section on page 12 (line 247-250): ‘Given these differences between participants with complete and incomplete data, we conducted MICE under the Missing at Random (MAR) assumption. To strengthen the plausibility of this assumption, both MMSE scores and fall history were included as predictors in the imputation model.’.*

Comment 4: What was the measurement scale of the outcome measure? Number of falls for instance is a count outcome measure and cost may be continuous. How did the authors analyze the count outcomes? Were they treated as continuous to fit Zellner's seemingly unrelated regression? Or were they treated as counts and fitted Poisson or negative binomial models?. I think more clarity must be provided regarding the measurement scale of the outcome measures, especially when in some cases is cost and in other cases is number of falls etc. The appropriate regression models must be applied in each case.

*Response and action: We treated the outcomes, including the number of falls and QALYs, as continuous variables to fit Zellner’s Seemingly Unrelated Regression. Our motivation for using SUR was its ability to account for the correlation between the error terms of cost and effect outcomes. While recognizing that the number of falls is a count outcome and QALYs as bounded continuous outcome, we argue that the use of Zellner’s SUR was justified for two reasons. First, the imputed data were bootstrapped to obtain reliable confidence intervals, thereby reducing reliance on strict distributional assumptions regarding the outcome measures. Second, the number of falls was separately analyzed using generalized linear models (GLMs) with appropriate distributional assumptions in the main effectiveness paper of the In Balance trial. These analyses yielded similar levels of uncertainty and did not alter the conclusions regarding intervention effects.*

*We included the sentence in the main analysis section on page 12 (line: 261 - 262): ‘To apply SUR, all outcomes (including the number of falls) were treated as continuous variables’. And we adjusted the sentence (line: 267 - 269): ‘To minimize reliance on distributional assumptions of continuous outcome scales, we estimated the uncertainty surrounding the ICERs using bias-corrected accelerated bootstrapping with 5,000 replications [36].’.*

Comment 5: Authors must clarify if they employed a robust standard error estimation in fitting the seemingly unrelated regression. The standard error estimation will affect the confidence intervals of the effect size estimates

*Response and action: We did not apply robust standard errors when calculating the 95% confidence intervals. Residual plots suggested homoscedasticity for the falls, injuries, and QALY outcomes. For costs, residuals did not show constant variance but also lacked a clear or systematic pattern (e.g., fan shape or curvature). Instead, we calculated the confidence intervals based on the variance of the resampled regression coefficients, providing a reliable estimate of uncertainty.*

*We adjusted the sentence in the main analysis section on page 12 (line 270 –271): ‘The 95% Confidence intervals were derived from the variance of the bootstrapped regression coefficients.’*

Comment 6: Authors must present results on complete case analysis as part of the sensitivity analysis to help determine how the results vary compared to pooled effect estimates from the multiple imputation by chained equations

*Response and action: We added a complete case analysis (CCA) as a sensitivity analysis. Compared to the imputed analysis, differences were mainly observed in the cost outcomes. Such variation was expected, as partially collected cost data could not be used in the CCA, resulting in a smaller sample size. Moreover, mean costs in the control group decreased relative to the intervention group, leading to a lower probability of cost-effectiveness. These results suggest that dropout from the trial was selective, with control group participants who had higher costs being more likely to drop out than those in the intervention group. In this regard, we deemed the analysis with MICE more reliable.*

*The results are presented in an extended version of Table 3. Additionally, we added or adjusted the following sentences. In the method section of the secondary analysis on page 13 (line: 280): ‘Furthermore, we conducted three sensitivity analyses to assess the robustness of the findings.’ and (lines: 291-293): ‘For the third sensitivity analysis (SA3), we conducted a complete case analysis to assess the robustness of the findings to the missing-at-random assumption and to explore the potential risk of bias due to missing data.’.*

*In the sensitivity analysis of the result section on page 21 (line: 392-396): ‘In SA3, between 90 and 100 participants with complete data were included, depending on the outcome measure. For the effect outcomes, the complete case analysis showed similar results compared to the main analysis. However, the difference in societal costs between the intervention and control group changed, with the difference in costs being closer to zero and the confidence intervals narrower (Table 3). This resulted in a lower probability of cost-effectiveness across all outcomes.’.*

*Lastly, in the discussion section on page 26 (line: 475-480): ‘The complete case analysis showed notable changes in the point estimates and uncertainty of costs compared to the main analysis. These results suggest that dropout from the trial was selective, with control group participants who had higher costs being more likely to drop out than those in the intervention group. We therefore preferred MICE, as it utilized more of the available data and likely prevented an underestimation of the cost difference between groups.’.*

On behalf of all authors,

Yours sincerely,

Judith Bosmans

**References**

1.	Folstein MF, Folstein SE, McHugh PR. “Mini-mental state”: A practical method for grading the cognitive state of patients for the clinician. Journal of Psychiatric Research. 1975;12(3):189-98.

2.	Hakkaart-van Roijen L VdLN, Bouwmans C, Kanters T, Tan S. Kostenhandleiding: Methodologie van kostenonderzoek en referentieprijzen voor economische evaluaties in de gezondheidszorg: Zorginstituut Nederland; 2024 [Available from: <https://www.zorginstituutnederland.nl/publicaties/publicatie/2016/02/29/richtlijn-voor-het-uitvoeren-van-economische-evaluaties-in-de-gezondheidszorg>.

3.	Faber MJ, Bosscher RJ, Paw MJCA, van Wieringen PC. Effects of exercise programs on falls and mobility in frail and pre-frail older adults: a multicenter randomized controlled trial. Archives of physical medicine and rehabilitation. 2006;87(7):885-96.

4.	Bull FC, Al-Ansari SS, Biddle S, Borodulin K, Buman MP, Cardon G, et al. World Health Organization 2020 guidelines on physical activity and sedentary behaviour. British journal of sports medicine. 2020;54(24):1451-62.

5.	Van Schooten KS, Van Dieen JH, Pijnappels M, Maier AB, van‘t Hul AJ, Niessen M, et al. The association between age and accelerometry-derived types of habitual daily activity: an observational study over the adult life span in the Netherlands. BMC public health. 2018;18(1):824.

6.	Van Schooten KS, Pijnappels M, Rispens SM, Elders PJ, Lips P, Daffertshofer A, et al. Daily-life gait quality as predictor of falls in older people: a 1-year prospective cohort study. PLoS one. 2016;11(7):e0158623.

7.	Hendriks MR, Evers SM, Bleijlevens MH, van Haastregt JC, Crebolder HF, van Eijk JTM. Cost-effectiveness of a multidisciplinary fall prevention program in community-dwelling elderly people: a randomized controlled trial (ISRCTN 64716113). International journal of technology assessment in health care. 2008;24(2):193-202.

8.	Peeters G, Heymans M, de Vries OJ, Bouter LM, Lips P, van Tulder MW. Multifactorial evaluation and treatment of persons with a high risk of recurrent falling was not cost-effective. Osteoporosis international. 2011;22:2187-96.

9.	Sherrington C, Fairhall N, Wallbank G, Tiedemann A, Michaleff ZA, Howard K, et al. Exercise for preventing falls in older people living in the community: an abridged Cochrane systematic review. British journal of sports medicine. 2020;54(15):885-91.

10.	White IR, Royston P, Wood AM. Multiple imputation using chained equations: issues and guidance for practice. Statistics in medicine. 2011;30(4):377-99.
